# The frequency of *efflux pump genes* expression in *Acinetobacter baumannii* isolates from pulmonary secretions

**DOI:** 10.1186/s13568-022-01444-4

**Published:** 2022-08-04

**Authors:** Ebrahim Rafiei, Milad Shahini Shams Abadi, Behnam Zamanzad, Abolfazl Gholipour

**Affiliations:** 1 Cellular and Molecular Research Center, Faculty of Medicine, Shahrekord University of Medical Sciences, Shahrekord, Iran; 2 Department of Microbiology and Immunology, School of Medicine, Shahrekord University of Medical Sciences, Shahrekord, Iran

**Keywords:** *Baumannii*, Antibiotic resistance, Efflux Pump, Tigecycline

## Abstract

*Acinetobacter baumannii* is an important opportunistic pathogen, and the cause of nosocomial infections worldwide in recent decades. Efflux pumps are considered as the important causes of multidrug resistance of *A. baumannii*. The aim of this study was to determine the frequency of efflux pump genes, and evaluate the antibiotic effect of Tigecycline on the expression of *adeB* gene in isolates of multidrug-resistant. *A. baumannii*. 70 isolates of *A. baumannii* were collected and confirmed by biochemical and molecular tests. Antibiotic resistance (Ciprofloxacin, Trimethoprim-sulfamethoxazole, and Tigecycline) was performed based on the minimum inhibitory concentration (MIC) method. Then, the effect of Carbonyl cyanide m-chlorophenyl hydrazone inhibitor (CCCP) on isolates was investigated and the frequency of *adeB, adeG, adeJ* and *abeM* genes were examined by PCR for isolates with reduced in MIC titer. Also, the antibiotic effect of Tigecycline on *adeB* gene expression in *A. baumannii* isolates was analyzed by Real-Time PCR. The antibiotic resistance for Ciprofloxacin, Trimethoprim-sulfamethoxazole, and Tigecycline was 97.1%, 95.8% and 37.2%, respectively. Following CCCP inhibitor use, the MIC titer had a decrease in MIC titer containing CCCP inhibitor was 64.3% for Ciprofloxacin, 51.5% for Trimethoprim-sulfamethoxazole and 50% for Tigecycline. The frequencies of genes associated with *adeB, adeG, adeJ* and *abeM* efflux pump were 100%, 92.8%, 86% and 98.5%, respectively. Real-Time PCR results showed a correlation between the antibiotic effects of Tigecycline on *adeB* gene expression. The antibiotic resistance of the isolates was relatively high. The isolates were resistant to Ciprofloxacin and Trimethoprim-sulfamethoxazole antibiotics, while more sensitive to Tigecycline. Also, efflux pump genes, which are the antibiotic resistance factors of *A. baumannii*, are frequently high in the isolates but it seems that isolates use other effluxe pumps than RND family to exit tigecycline.

**Graphical Abstract:**

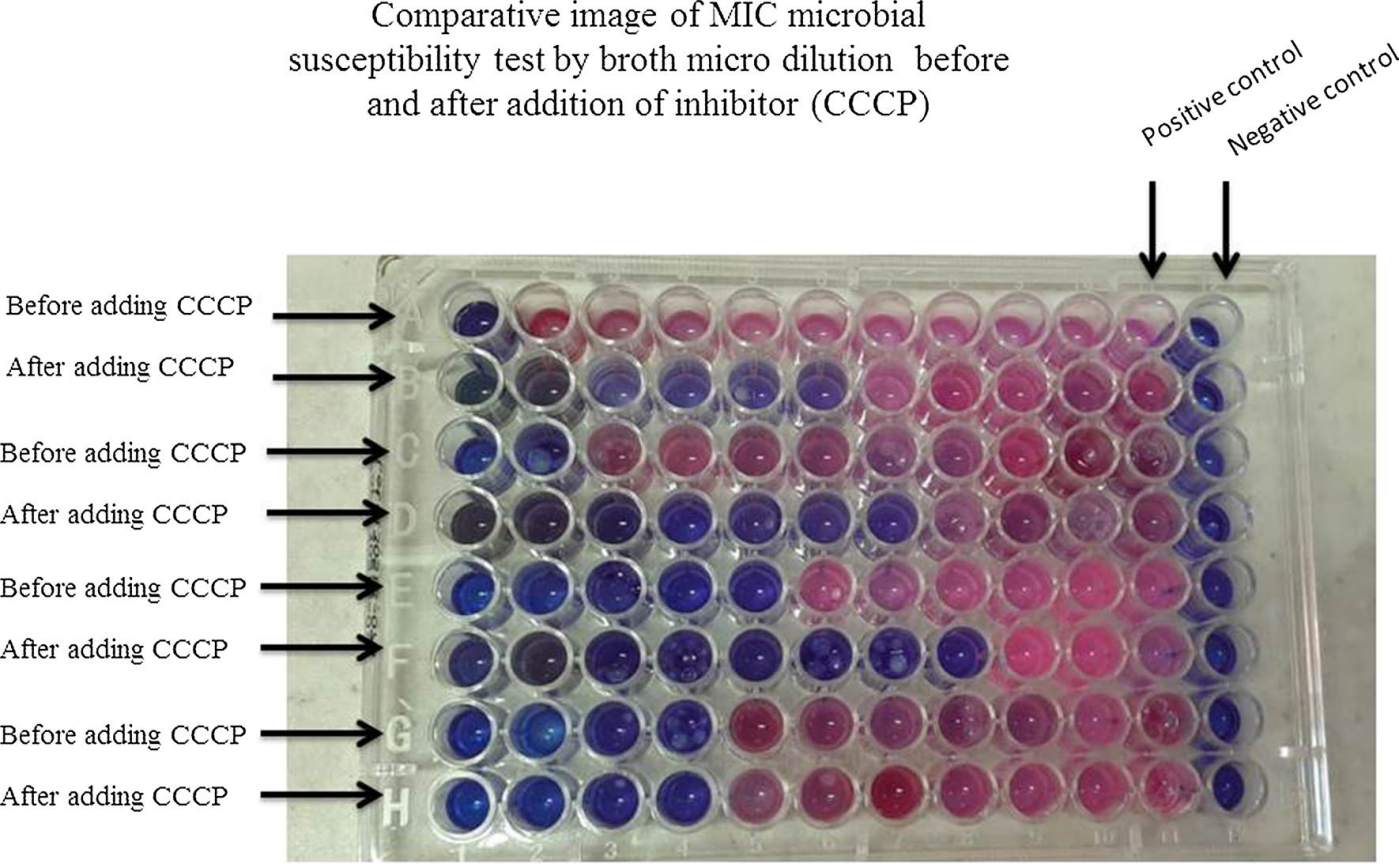

## Key points

- The frequencies of genes associated with adeB, adeG, adeJ and abeM efflux pump were 100%, 92.8%, 86% and 98.5%, respectively.
- Real-Time PCR results showed a correlation between the antibiotic effects of Tigecycline on adeB gene expression. The antibiotic resistance of the isolates was relatively high.
- Also, efflux pump genes, which are the antibiotic resistance factors of A. baumannii, are frequently high in the isolates but it seems that isolates use other effluxe pumps than RND family to exit tigecycline.

## Introduction

*Acinetobacter baumannii* is an aerobic gram-negative coccobacillus that causes nosocomial pneumonia, bloodstream infections, meningitis, and urinary tract infections (Pan et al. 2016). The prevalence of multidrug resistance in *A. baumannii* isolates in Iran has increased from 50% in 2001 to 2007 to 74% in 2010 to 2015 (Bialvaei et al. 2017). Patients in the intensive care unit (ICU), and dependent ventilator in particular, are at potential risk of this infection. The high mortality rate accounted for patients with weakened immune systems in ICU. The treatment of *A. baumannii* infection is greatly difficult due to the high epidemic potential, formation of a biofilm in the external body sites, and multi-drug resistance (MDR) (Liu et al. 2016). Various studies have reported that *A. baumannii* clinical isolates are resistant to antimicrobial agents such as Aminopenicillins, Uridopenicillins, broad-spectrum Cephalosporins, most Aminoglycosides, Quinolines, chloramphenicol, and Chloramphenicol (Antunes et al. 2014; Towner 2009).

The underlying molecular mechanisms for multiple drug-resistant (MDR) of *A. baumannii* are related to the changes in membrane permeability and antibiotic target proteins, as well as the production of β-lactamase enzymes, and multidrug efflux pumps(Wang et al. 2014). Efflux pumps play a role in endogenous and acquired resistance in *A. baumannii* to a variety of antibiotics and antiseptics. Bacterial efflux systems reduce the intra-accumulation of antibiotics leading to the increase of minimum inhibitory concentration (MIC) (Liu et al. 2018). The efflux system of *A. baumannii* includes resistance nodulation division superfamily (RND), and multidrug and compound extrusion family (MATE) (Coyne et al. 2011). RND pumps such as Acinetobacter drug efflux (Ade) adeB, adeG, adeJ efflux antibiotics, biocides and antiseptic substances via ATP (Abadi et al. 2018) (Xu et al. 2019). Efflux pumps adeB, adeG, adeJ have been reported in *Acinetobacter* species (Kaviani et al. 2020). In addition, MATE pump, like abeM, effluxs Fluoroquinolones and Imipenem using sodium ion gradient (Xu et al. 2019). In regard to increasing MDR in *A. baumannii* infection, the use of inhibitors that restore the bacterial sensitivity to antibiotics by inhibiting efflux pumps is needed (Sharma et al. 2019). Efflux pump inhibitors are compounds that block the outpour activity of pumps by competitive and non-competitive methods with substrate. Carbonyl cyanide 3-chlorophenylhydrazone (CCCP) degrades the electrochemical gradient of proton membrane ions by separating phosphorylation from oxidation. This compound can suppress the function of efflux pumps in *A. baumannii* through disturbing the proton-motive force and may reduce the level of MDR (Moazzen et al. 2018).

The aim of this study was to determine the frequency of efflux pump *adeB*, *adeG*, *adeJ* and *abeM* genes, and evaluation of the antibiotic effect of Tigecycline on the expression of *adeB* gene in isolates of *A. baumannii*, which were resistant to multiple drugs.

## Materials and methods

Samples were obtained from patients hospitalized in the ICU Unit of Kashani Hospital, Shahrekord, Iran over a period of 6 months from 2019. All the clinical samples were obtained during the routine procedure of medical care.

### Patients and inclusion criteria

We collected 70 isolates from the respiratory secretions of patients. All isolates were transferred to the Cellular and Molecular Research Center. Biochemical tests of fermentation and H_2_S on triple sugar iron (TSI) agar medium, motility and indole on SIM medium, growing on MacConkey agar medium at 37 and 42, and oxidase/catalase tests were carried out to identify *A. baumannii* isolates. To confirm *A. baumannii* species, *blaOXA-51* gene was detected (by primer sequences: F: 5′-TAA TGC TTT GAT CGG CCT TG-3′; R: 5′-TGG ATT GCA CTT CAT CTT GG-3′) in isolates. Immune disease background and prior hormone therapy were recognized as the exclusion criterion.

### Genomic DNA extraction and PCR

To extract DNA, several colonies of the desired isolates were firstly dissolved in 100 μL of sterile distilled water in the 1.5 mL microtubes. Then, the microtubes were placed in a hot plate (95 °C) for 15 min, and centrifuged for 10 min at 14,000 rpm. The supernatant containing DNA was transferred to a 0.5 mL microtube for PCR reaction. To determine the purity of the extracted DNA, absorption at 260/280 nm was the ratio between 1.8 and 2.

The PCR was performed for *adeB, adeG, adeJ* and *abeM* genes using the specific primers (Table.1). The PCR program included a denaturation step for 5 min at 95 °C, with 30 denaturation cycles for 45 s at 95 °C, annealing for 60 s at 58 °C, and extension for 60 s at 72 °C, with a final extension for 5 min at 72 °C. The products from the PCR were electrophoresed in the 1% agarose gel.

**Table 1:** The primers used for PCR

Number	Gene	Sequence primer 5'–3'	Length of product (bp)
1	adeB-F	AACGGACGACCATCTTTGAG	84
2	adeB-R	CAGTTGTTCCATTTCACGCA	
3	adeG-F	CCAGACAAAGCCCAACAA	769
4	adeG-R	AAATAACGACCACAACAACC	
5	adeJ-F	ATTGCACCACCAACCGTAAC	463
6	adeJ-R	TAGCTGGATCAAGCCAGATA	
7	abeM-F	AAGTCTTTATTGCCGCACAC	361
8	abeM-R	ATCGGTGCCTGAGTATCTTG	
9	16S-F	CAGCTCGTGTCGTGAGATGT	112
10	16S-R	CGTAAGGGCCATGATGACTT	

### Determination of the minimum inhibitory concentration

The minimum inhibitory concentrations (MICs) of three antibiotics including Ciprofloxacin, Trimethoprim-sulfamethoxazole, and Tigecycline for *A. baumannii* were determined by the broth microdilution method according to the Clinical and Laboratory Standards Institute (CLSI) (Kaviani et al. 2020). Positive control wells included culture medium and bacterial suspension, and negative control wells included culture medium and antibiotics. Pseudomonas aeruginosa ATCC 27853 standard strain was used as the positive control.

To detect isolates containing efflux pumps, 0.25 µg/mL Carbonyl cyanide 3-chlorophenylhydrazone (CCCP) was added to broth medium in the separate micro plate, and MIC was evaluated. Finally, by comparing the MICs before and after adding CCCP efflux pump inhibitor, the isolates with titer reduction were considered as isolates with efflux pump.

### RNA isolation and RT-PCR

To examine *adeB* gene expression, isolated were treated with MIC and sub MIC of CCCP*.* RNA from each isolate was extracted using RNX- Plus reagent (SinaClon BioScience; Iran) solution according to the manufacturer’s instructions. The Thermo Scientific^™^ NanoDrop 2000 calculated the concentration and purification of each RNA sample. cDNAs were synthesized using the Revert Aid first-strand cDNA synthesis kit (Yektatajhiz; Iran) with 1.5 μg of RNA at a 20 μL reaction rate. The Real-Time PCR was conducted on a Rotor-Gene RG-300 (Corbett Research, Sydney, AU) and SYBR Green Real-time PCR Master Mix kit (Yektatajhiz; Iran) to show the quantification of mRNA. A paired primer was applied for the amplification of *adeB* gene (primer sequence: F: 5'-AACGGACGACCATCTTTGAG-3'; R: 5'-CAGTTGTTCCATTTCACGCA-3'). Thermal cycling continued the denaturation process at 95 °C for 5 min for the first denaturation stage at 95 °C for 5 min and then 38 cycles at 95 °C for 15 s, 61 °C for 20 and 72 for 25 s. The amplification specificity of *adeB* gene expression was confirmed by the melting curve. *adeB* gene expression was standardized against 16sRNA as an internal control, and relative quantification (2^−ΔΔCq^) revealed the fold changes of expression.

### Statistical analyses

All the data analyzed in this study were anonymized. The mean ± standard deviation was viewed as results. Comparisons between positive and negative genes and *abeM* gene expression were calculated using *t*-tests and χ^2^tests. Drug resistance was assessed by logistic regression for analyzing the odds ratio after adjustment for gender, mean age. Differences were found statistically meaningful for *P*-values less than 0.05. The data were analyzed using SPSS version 24.

## Results

### Specimen collection and culture

In this study, there were 56 men and 14 women with mean age of 58.16 years. All 70 isolates were identified as *A. baumannii* by biochemical differential tests. Also, *blaOXA-51* gene PCR confirmed the isolation of *A. baumannii*.

### Pattern of antibiotic resistance

MIC microdilution method was used to determine antibiotic resistance, and Ciprofloxacin, Trimethoprim-sulfamethoxazole, and Tigecycline antibiotics were evaluated in this study. As can be observed in Table.2, the antibiotic resistance rate for Ciprofloxacin (S ≤ 1, I = 2, R ≥ 4 µg/mL), Trimethoprim-sulfamethoxazole (S ≤ 2/38, R ≥ 74.4 µg/mL), and Tigecycline (S ≤ 1, R ≥ 2 µg/mL) were measured according to the US CLSI criteria. The results showed that the highest antibiotic resistance accounted for Ciprofloxacin, and Trimethoprim-sulfamethoxazole, and Tigecycline were in the next level of antibiotic resistance respectively.

**Table 2:** Antibiotic resistance pattern before using CCCP pump inhibitor

Antibiotic	Resistance	Semi sensitive	Sensitive
Ciprofloxacin	(97/1) 68	(2/9) 2	(0) 0
Trimethoprim-sulfamethoxazole	(95/8) 67	(0) 0	(4/2) 3
Tigecycline	(2/37) 44	(0) 0	(63/8) 26

The results of the antibiotic resistance test by the MIC method indicated that 50 isolates contained *A. baumannii* with efflux pump (Table 3).

**Table 3:** Antibiotic resistance pattern after using CCCP pump inhibitor

Antibiotic	Changed MIC	Unchanged MIC
Ciprofloxacin	45 (64.3)	25 (35.7)
Trimethoprim-sulfamethoxazole	36 (51.5)	34 (48.5)
Tigecycline	35 (50)	35 (50)

### PCR results for adeB, adeG, adeJ and abeM genes

After identifying isolates with efflux pump (50 isolates), the frequency of *adeB, adeG, adeJ* and *abeM* genes was examined by PCR on these isolates. The results showed that the frequency of *adeB, adeG, adeJ* and *abeM* genes in *A. baumannii* isolates were 100%, 90%, 86%, and 98% respectively.

### The effect of Tigecycline antibiotic on adeB gene expression

**Figure 1:**
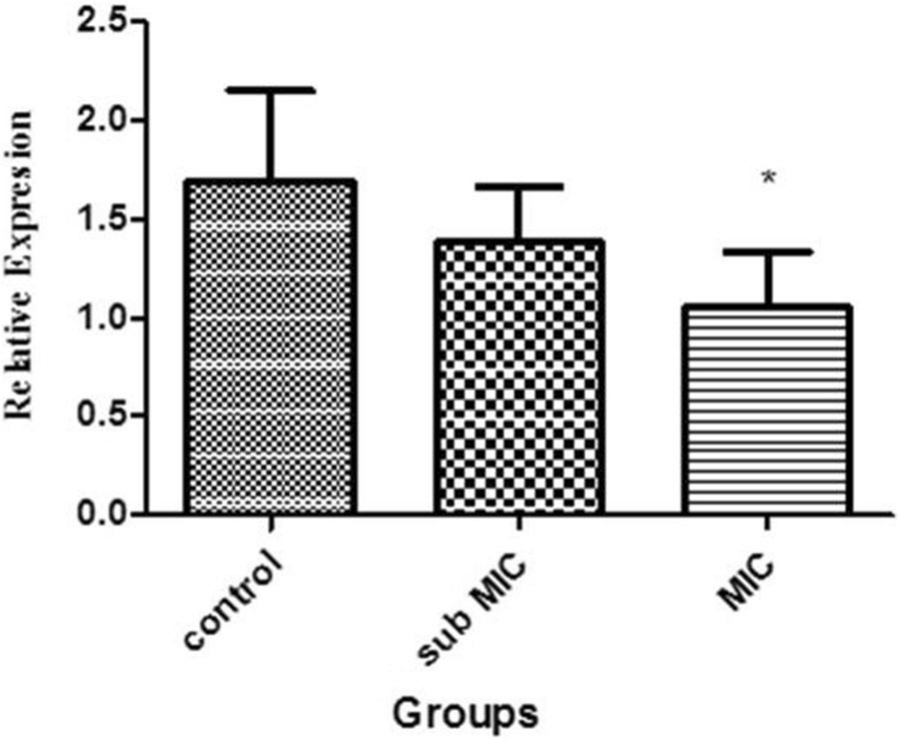
Real-Time PCR was used to analyze the *adeB* gene expression in *A. baumannii* isolates. A significant decreased in *adeB* gene expression was observed in MIC concentrations

The effect of Tigecycline on *adeB* gene expression was evaluated using Real-Time PCR in *A. baumannii* isolates at MIC concentrations. The resulst showed that CCCP resulted in the reduction of *adeB* gene expression that difference at the doses of MIC was significant compared to the control group (Fig. 1).

## Discussion

*A. baumannii* is a gram-negative, aerobic, non-fermentative and oxidase-negative coccobacillus. This opportunistic bacterium is one of the most important causative agents of nosocomial infections that can be isolated from blood, sputum, pleural fluid and urine samples of patients. *A. baumannii* is involved in nosocomial infections such as endocarditis, meningitis, pneumonia, burns and sepsis (Bergogne-Bérézin and Towner 1996). One of the unique features of *A. baumannii* is resistance to antibiotics and acquiring resistance genes to antibiotics, which exacerbate the treatment in patients with the deficient immune system. Recently, *A. baumannii* strains with the resistant ability to all common antibiotic classes are undergone prevalence in healthcare systems (Moammadi et al. 2014). ICU accounts for only about 5–15% of hospital beds, but more than 30% of nosocomial infections are related to these sections. *A. baumannii* is resistant to most antibiotics, such as aminoglycosides, fluoroquinolones, beta-lactams, and cephalosporins, presenting more challenges for treatment (Amini et al. 2016).

In this study, 70 isolates of *A. baumannii* were collected from the pulmonary secretions of patients admitted to the ICU of Kashani Hospital of Shahrekord. We investigated the pattern of antibiotic resistance of these isolates against the Ciprofloxacin, Trimethoprim-sulfamethoxazole, and Tigecycline antibiotics. The resistance to Ciprofloxacin, Trimethoprim-sulfamethoxazole, and Tigecycline antibiotics in this study were 97.1%, 95.8% and 37.3% respectively. A meta-analysis study carried out in 2017 indicated 87 ± 12 antibiotic resistance rate to Ciprofloxacin in 11 hospitals (Hidron et al. 2008). Afshari et al. examined the pattern of antibiotic resistance for *A. baumannii* isolates from patients referred to the ICU section. The resistance of the isolates to Ciprofloxacin was 94% and the resistance to Tigecycline was 92% (Yavari et al. 2016). In 2017, Kia et al*.* evaluated the frequency of antibiotic resistance of *A. baumannii* in clinical isolates from respiratory secretions, urine, blood and body fluids of patients in ICU, surgical, infectious, orthopedic and burn sections in Kerman. In this study, the resistance to Ciprofloxacin and Tigecycline was reported to be 98% and 36% respectively (Emami et al. 2018).

The activation of efflux pumps has been proposed as an intrinsic mechanisms of antibiotic resistance in bacteria (Vila et al. 2007). The frequency of *adeB, adeG, adeJ* genes from RND family and *abeM* gene from MATE family were analyzed by PCR. RND and MATE families are classified as the efflux pumps in *A. baumannii* that maximize the concentration of MIC. RND family efflux pumps play a crucial role in the development of antibiotic resistance in *A. baumannii*. Three types of RND family, including AdeABC, AdeIJK, and AdeFGH efflux pumps, have been identified (Magnet et al. 2001). The frequency of *adeB, adeG adeJ* and abeM in our study were 100%, 90%, 86% and 98%, respectively. In 2019, the prevalence of efflux pump genes was examined among 154 isolates of *A. baumannii* in Korea. The frequency of *adeG, adeB, adeE, adeY, abeM* and *adej* genes were 98.7%, 89%, 13.6%, 7.7%, 95.5% and 98.9% respectively (Choi et al. 2019). In a similar investigation, the frequency of *adeA, adeB, adeC, AbeM*, and *adeS* genes in isolates of multidrug-resistant *A. baumannii* isolated from patients admitted to Alzahra Hospital in Isfahan was evaluated as 100%, 96%, 95%, and 8, respectively. From 80 *A. baumannii* isolates of this study, 39 isolates had a MIC higher than 32 μL and were resistant to Amikacin, 41 isolates with a MIC greater than 2 μL. Following the combination of CCCP inhibitor, 25 isolates had a twofold decrease and 15 isolates had a fourfold decrease in the MIC of Amikacin (Ostadi et al. 2019). The difference in the frequency of genes in various studies is in consist with geography and epidemiology of these areas. However, the results of the frequency of efflux pump genes in the present study are similar to most studies, and indicate the high frequency of efflux pump genes in *A. baumannii* isolates from pulmonary secretions.

Additionally, CCCP inhibitor was used to identify efflux pumps in *A. baumannii* isolates. After using CCCP, the MIC changes of the isolates were 64.3% for Ciprofloxacin, 51.5% for Trimethoprim-sulfamethoxazole and 50% for Tigecycline. The CCCP compound with inhibitory capacity of cytochrome oxidase blocks the electron transfer chain at position 3. Interruption of the electron transfer chain causes phosphorylation to separate from oxidation and destroys the electrochemical gradient of proton ions in the membrane (JaponiNejad 2014). Thus, the CCCP can inhibit efflux pumps, including efflux pumps of *A. baumannii* species, which use proton-driven force to remove antimicrobials from the cell and reduce the level of drug resistance in bacteria (Piddock and Johnson 2002). Studies have shown that efflux pumps increase the MIC of the antibiotics Ofloxacin, Ciprofloxacin and Gentamicin in *A. baumannii* (Basatian-Tashkan et al. 2020). A study by Hornsey et al*.* in 2010 demonstrated a rise in the MIC of Tigecycline by increasing the expression of adeABC efflux pumps (Patel et al. 2010). In the results of Ardabili et al*.*, all strains of *A. baumannii* were resistant to Ciprofloxacin, at about 4–128 µg/mL, in MIC measurement. Likewise, they showed that the resistance of the strains to Ciprofloxacin decreased 2–64-fold in the presence of the CCCP efflux pump inhibitor in 86% of the strains (Mahamoud et al. 2007). In 2006, a significant reduction in the MIC of antibiotic-resistant strains using the CCCP inhibitor was reported. The observations of this study also indicated that drug resistance in clinical isolates was mediated by an active efflux pump that transports the accumulated drug to the outside (Abdi-Ali et al. 2006). Beheshti et al. have reported the role of efflux pumps in the resistance of *A. baumannii* isolates to Tetracycline antibiotics. In this study, by adding CCCP inhibitor, a decrease in MIC in tetracycline-resistant *A. baumannii* isolates was observed, which decreased 2–4 folds in 18 isolates, 8 folds in 26 isolates, 16 folds in 1 isolate, and 32 folds in 1 isolate (Beheshti et al. 2014). Comparison of the results of the present study with other studies shows that efflux pumps have been effective in the development of antibiotic resistance in *A. baumannii* isolates. The difference between the results of isolates with an effusion pump in this study and the results of other studies is due to the diversity of samples used in those studies compared to the present study.

Furthermore, the effect of Tigecycline on the expression of *adeB* gene was examined in the isolates containing *abeM* gene. The relationship between increased *adeB* gene expression and its association with increased resistance to Tigecycline in *A. baumannii* has been reported in Yang et al.’s study (Yang et al. 2019). 61% of isolates with antibiotic resistance in a study in Egypt were prone to a significant increase of *adeB* gene expression compared to sensitive isolates (Aladel et al. 2020). Ardehali et al. in their study found the association between *adeJ* gene expression and its association with increased resistance to Tigecycline in *A. baumannii* (Ardehali et al. 2019). Although an increase in the expression of *adeB* gene has been reported in antibiotic resistance *A. baumannii* isolates, the expression of *adeB* gene in our study showed a decrease in the presence of the antibiotic Tigecycline. In regard to the various mechanisms of antibiotic resistance, the development of antibiotic resistance of *A. baumannii* isolates should also be considered. On the other hand, it is possible that the bacterium expressed other genes in the RND family when exposed to antibiotic treatment. It should also be noted that the mere expression of efflux pump genes in isolates does cause resistance. The expression of RND family genes is controlled by other regulatory genes. Any mutation in these regulatory genes affects the expression of genes under their control. According to the results of this study and previous studies, various strains of *A. baumannii* seem to use efflux pump for antibiotics resistance.

Antibiotic resistance among *A. baumannii* isolates can make efforts to introduce inhibitors of efflux pumps along with antibiotics in order to inactivate the activity of efflux pumps and prevent the development of antibiotic resistance. Based on the Real time PCR results it seems that isolates use other effluxe pumps than RND family to exit tigecycline. It is also suggested that due to the frequency of efflux pumps in the studied isolates, the expression of other genes of RND family such as *adeG* and *adeJ* genes of efflux pump as well as *abeM* gene of MATE family which are pumps in *A. baumannii* should be studied in future studies.

## Data Availability

The data are available. All data generated or analyzed during this study are included in this study.

## Abbreviations

MIC: Minimum inhibitory concentration
CCCP: Carbonyl cyanide m-chlorophenyl hydrazone inhibitor
ICU: Intensive care unit
MDR: Multi-drug resistance
RND: Resistance nodulation division superfamily
MATE: Multidrug and compound extrusion family
Ade: *Acinetobacter* drug efflux
TSI: Triple sugar iron
PCR: Polymerase chain reaction
